# When failure should be the option

**DOI:** 10.1186/1741-7007-8-61

**Published:** 2010-05-21

**Authors:** Gregory A Petsko

**Affiliations:** 1 Rosenstiel Basic Medical Sciences Research Center, Brandeis University, Waltham, MA 02454-9110, USA

## 

Give me your tired, your poor, your Phase II failures... Well, OK, to be honest, I'm not sure I want your tired or your poor, and besides, The Statue of Liberty has that pretty well covered. But I am sure that I want your Phase II failures. I REALLY want your Phase II failures.

Before I explain what I mean, I should review the progress in making drugs to treat serious human illnesses in the first decade of the 21st century - or, more accurately, the lack of progress. While it's been true for a long time that about the hardest thing human beings have ever tried to do is to make a drug, it seems as though lately it's got even harder. The number of new therapeutics approved for use on humans, per year, has been essentially flat for more than two decades. During this time, new technologies such as structure-based drug discovery have been created, pharmaceutical companies have merged to form giant entities, and hundreds of biotechnology companies have been launched to rival them, the research expenditure of the National Institutes of Health (NIH) has more than doubled, and the R&D budget of the drugs industry has shot up. Yet despite all that, we are not developing new drugs any faster than we did before. Why not?

One reason is that the diseases we are now trying to treat, such as cancer and Alzheimer's disease, are harder than many of the infectious diseases that dominated our efforts 50 years ago. But another reason lies in the nature of the drug development process itself.

For every drug that is used clinically to treat a disease, more than 6,000 completely new chemical compounds are synthesized. On average, about 20 drug candidates are tested in people for every one that gets to market. (The failure rate for biopharmaceuticals, which are macromolecules rather than small organic compounds, is a lot better, but still very high: about one in five of those candidates tested in humans make it to the clinic.) For chemical drugs the process takes, on average, about 12 years from target identification to drug approval and costs close to US$1 billion (the timeline is shorter for biopharmaceuticals and the overall cost is only about a third of this, in part because there are fewer failures). More than half the cost comes from the clinical trials that must be undertaken once a drug candidate has been approved for human testing. This approval comes at the end of an extensive period of preclinical testing, which involves *in vitro *(test tube) and *in vivo *(animal or cell culture) experiments using wide-ranging doses of the study drug to obtain preliminary efficacy, toxicity and pharmacokinetic data (data on absorption, distribution, metabolism and excretion, popularly known as ADME) in animal models of the disease in question. Such tests allow pharmaceutical companies to decide whether a drug candidate warrants further development as an investigational new drug.

In the United States, which is both the largest drug maker and the largest single drug market in the world, the next step is the clinical trial process, which has three main phases. Phase I trials are the first stage of testing in human subjects. Normally, a small (20 to 100) group of healthy volunteers is selected. They are given the drug to assess its safety, tolerability, pharmacokinetics and pharmacodynamics in people. These trials are often conducted in an in-patient clinic, where the subject can be observed by full-time staff. Phase I trials also include dose-ranging, also called dose escalation, studies so that the appropriate dose for therapeutic use can be estimated. The tested range of doses will usually be a fraction of the dose that caused harm in the preclinical animal testing. Although Phase I trials typically use healthy human volunteers, there are circumstances in which real patients are used, such as patients who have terminal cancer or are infected with HIV and lack other treatment options.

Once the initial safety of the drug candidate has been confirmed in Phase I trials, Phase II trials are performed on larger groups (20 to 300), this time of patients. Phase II trials are designed to assess how well the drug works on the disease in question, as well as to continue Phase I safety assessments in a larger group of volunteers and patients. Phase II studies are sometimes divided into Phase IIA and Phase IIB, where Phase IIA is specifically designed to assess dosing requirements (how much drug should be given) in patients, and Phase IIB is specifically designed to study efficacy (how well the drug works at the prescribed dose(s)).

Phase III studies are randomized, controlled, multicenter trials on large patient groups (usually 300 to 3,000 or more depending upon the disease/medical condition studied) and are aimed at being the definitive assessment of how effective the drug is in comparison with current 'gold standard' treatment for the disease. Because of their size and comparatively long duration, Phase III trials are the most expensive, time-consuming and difficult trials to design and run, especially in therapies for chronic medical conditions. While not required in all cases, it is typically expected that there be at least two successful Phase III trials in order to obtain approval from the appropriate regulatory agencies such as the Food and Drug Administration (FDA) (in the United States), or the European Medicines Agency (EMEA) (in the European Union), for example. Once a drug has proved satisfactory after Phase III trials, the trial results are usually combined into a large document containing a comprehensive description of the methods and results of all human and animal studies, the manufacturing procedures, formulation details, and shelf life. This collection of information makes up the 'regulatory submission' that is provided for review to the regulatory agencies. They will review the submission and make the final decision on whether to grant the pharmaceutical or biotechnology company approval to market the drug. However, many drugs undergoing Phase III clinical trials can be marketed under FDA norms with proper recommendations and guidelines (after all, they have been shown to be both safe and effective in the Phase I and Phase II studies), but in case of any adverse effects being reported anywhere, the drugs will be recalled. While most pharmaceutical companies refrain from this practice, it is possible to see drugs that are still undergoing Phase III clinical trials being used in the clinic.

So, given that the failure rate for small-molecule therapeutics is about 19 out of 20 once clinical trials commence (and 4 out of 5 for biologicals), where do you think the major roadblock is to approval? If you said Phase I, because that is where toxicity (and side-effects) are first assessed, you said what I once thought, and you're wrong. It's not in Phase III either; in fact, although failures in Phase III do occur (and often engender a lot of publicity), most drugs that enter Phase III trials are eventually approved. The bottleneck is in Phase II.

That's right: after years of research and hundreds of millions of dollars, the majority of drugs that never get to market fail because they do not show efficacy in the disease they were intended for. That's a staggering fact, and it has a number of important implications. One of the implications is that our animal models for toxicity are pretty good (after all, the Phase II failures passed Phase I, which looked for toxicity), but our animal and cell-culture models for disease are very poor for many diseases. In other words, we lack good models that would allow us to validate targets and fail compounds much earlier in the drug-development pipeline, when the cost would be much lower. If I were the pharmaceutical and biotech companies, I would be funding a lot of research in academic labs on the development of better disease models (induced pluripotent stem cells (iPS cells) may be a big advance here, but it's too early to tell), and I would be working more closely with academic labs for longer periods of time to validate targets and find additional targets deeper into the development process.

But that isn't my main point. My main point is that the Phase II failures represent an enormous, untapped resource for the biomedical sciences - a resource that could go a long way towards solving the problem of low productivity, in terms of cures, that plagues both industry and academic medicine.

You see, the Phase II failures have all passed Phase I, so they have been shown to be safe in humans. They failed for efficacy. They failed because they did not effectively treat the disease they were intended to treat, even though they showed biological activity in assays and model systems. There are hundreds of them - perhaps more than a thousand. I don't know the number because drug companies bury those failures. They don't want to release a lot of information about the molecules in question because, among other things, they fear that will give their competitors too much of an insight into what they are working on. But here's the question I would like you - and them - to ponder. What if those drugs were not tried on the right disease?

We now know that many quite different diseases share common pathways and processes in the cell. Cancer is a disease of abnormal cell survival; in Alzheimer's disease the survival pathways have failed. Alzheimer's patients have significantly lower risk of many cancers. What if the cure for Alzheimer's disease is sitting on some drug company's shelf, as a potential cancer drug that failed in Phase II? (A biotech company called Link Medicines is currently testing one such failure to find out.) Gaucher disease and Parkinson's disease both involve lysosomal damage and display aggregates of a protein called alpha-synuclein; Gaucher carriers are at elevated risk for Parkinson's. What if a drug intended to cure Gaucher disease, one that failed in Phase II, is actually a treatment for Parkinson's? (Another biotech company, Amicus Therapeutics, is beginning to investigate that possibility.) Recent studies show that people diagnosed with psoriasis are at greater risk of developing heart disease; in fact, in patients with severe psoriasis who are younger than 50 years old, the risk is comparable to that seen in diabetes. How many Phase II-failed psoriasis drugs have ever been tested in heart disease clinical trials?

I could list literally a dozen more examples, but you get the idea: because we balkanize biomedical research, biomedical-research funding, and pharmaceutical industry drug development according to phenotypically classified diseases, the possibility that a drug that has failed the efficacy test in one disease might be efficacious in a completely different one has not permeated the culture. Yet we should not be surprised if such cross-disease activity occurs, because these Phase II failures got as far as human clinical trials for a reason: they hit a target (or targets) in cell culture and animal models, and produced an effect. That they failed to do so in the human disease is an indictment of our disease models, not the biochemical and cellular data that showed they did something. What we need to do is test them on other diseases - a battery of other diseases - perhaps first in cell culture (iPS cells?) and animal models and, if they show an effect, then directly in Phase II clinical trials for the new disease. (It may even be that we can proceed immediately to Phase II studies without the animal model testing (after all, our animal models aren't very good) if we have mechanistic or other data to suggest that efficacy is possible in the unrelated disease.) The problem is that such tests usually cannot be done by the original developer, because no drug company has programs in all the major human diseases. And they certainly aren't going to let their competitors test them. So who will do the tests?

Academic labs are the perfect answer. In most cases, they discovered the disease targets and developed the disease models in the first place. Many of these labs are already trying to find compounds that show efficacy in those models, in the hope that a pharmaceutical or biotech company will become interested in developing them further. But they lack libraries of compounds to test that are known to be safe in humans and that are guaranteed to interact with something, anything, in the cell. The Phase II failures are a perfect library to test.

If the tests are successful, who would take the next steps of funding the new Phase II clinical trials? It makes sense for such funding to come from the government, and there is a new program that might be an interesting way to do it. In the new health-care reform bill recently signed into law by US President Barack Obama, there is an amendment that authorizes the NIH to establish a $500 million a year program called the Cures Acceleration Network, whose mission is to aid in establishing partnerships between academic labs and industry that would accelerate the finding of cures for untreatable human illnesses. I have discussed this amendment in more detail in an article in *Genome Biology *[1]. At present, there is no agreement on just how to fulfill that charge. I think finding new indications for some of the Phase II failures would be a great way to do that. Since they already have passed Phase I, they are very much closer to approval than any set of random compounds from other libraries, or even compounds that are currently in preclinical testing; all that is needed is to find the right disease for them, if one exists. $500 million per year would fund a number of Phase II clinical trials, as well as a grant program to identify academic labs to test the compounds in disease models for those cases where a second disease indication is not obvious from the biology. If efficacy is established against a new disease in humans, then the government could give the company that originally developed the compound the right of first refusal on an option to fund Phase III trials and then to market the drug. If that company was not interested, the government could hold a competition to select another company that would take the compound forward. In any case, the academic lab that established the new disease indication would get some royalties from the sales, as would the original developer.

Why should pharmaceutical companies be interested, given how jealously they guard their secrets? After all, the probability that one of their compounds will show any efficacy in a new disease is still quite low. However, there are some incentives, including especially good publicity, that might be persuasive - after all, the pharmaceutical industry has taken a big beating lately in the court of public opinion. It also might be possible to find a legislative fix for some of their problems that could be traded for their participation in a Phase II failures program. And steps could certainly be taken to protect the confidentiality of some of the information about the compounds in question, at least for a time. We can find the right incentives if we try.

So I want the Phase II failures. I REALLY want the Phase II failures. I want them for my own research and for your research. I want them because they could make a difference for a host of unmet medical needs. And here's my last question, aimed at patient advocacy groups and scientific societies and medical school deans and biotechnology associations and government officials everywhere: who wants to help me get them?
